# Time perception in bipolar disorder: a systematic review

**DOI:** 10.1017/neu.2024.57

**Published:** 2025-01-23

**Authors:** Andrea Escelsior, Maria Bianca Amadeo, Alberto Inuggi, Margherita Guzzetti, Yara Massalha, Alice Trabucco, Giacomo Marenco, Beatriz Pereira da Silva, Monica Gori, Georg Northoff, Mario Amore, Gianluca Serafini

**Affiliations:** 1 IRCCS Ospedale Policlinico San Martino, Genoa, Italy; 2 Department of Neuroscience, Rehabilitation, Ophthalmology, Genetics, Maternal and Child Health (DINOGMI), Section of Psychiatry, University of Genoa, Genova, Italy; 3 U-VIP Unit for Visually Impaired People, Fondazione Istituto Italiano di Tecnologia, Genoa, Italy; 4 Department of Pathophysiology and Transplantation, University of Milan, Milan, Italy; 5 Mind, Brain Imaging and Neuroethics Research Unit, The Royal’s Institute of Mental Health Research, University of Ottawa, Ottawa, ON, Canada

**Keywords:** Time perception, bipolar disorder, mania, depression, time

## Abstract

**Objective:**

Time distortions characterise severe mental disorders, exhibiting different clinical and neurobiological manifestations. This systematic review aims to explore the existing literature encompassing experimental studies on time perception in patients with bipolar disorder (BD), considering psychopathological and cognitive correlates.

**Methods:**

Studies using an experimental paradigm to objectively measure the capacity to judge time have been searched for. Selected studies have been described based on whether i) explicit or implicit time perception was investigated, ii) the temporal intervals involved were sub-second or supra-second, and iii) a perceptual or motor timing paradigm was used.

**Results:**

Only 11 met the criteria for inclusion in the review. The available literature shows that the performance of BD patients mostly aligns with controls within sub-second timeframes (six articles), while a different pattern emerges within supra-second intervals based on the clinical phase of the disease (seven articles). Specifically, for longer temporal spans, BD patients tend to overestimate the duration during manic states and underestimate it during depressive states. Notably, no studies have directly investigated the neurobiological mechanisms associated with time perception.

**Conclusion:**

This review indicates that BD patients exhibit time perception similar to controls within sub-second intervals, but tend to overestimate time and underestimate it based on the clinical phase within supra-second intervals. Expanding the understanding of time perception in BD, particularly in relation to clinical phases and cognitive function, is of great importance. Such insights could deepen our understanding of the disorder, refine diagnostic processes, and guide the development of innovative therapeutic interventions.

## Graphical abstract

**Figure:**
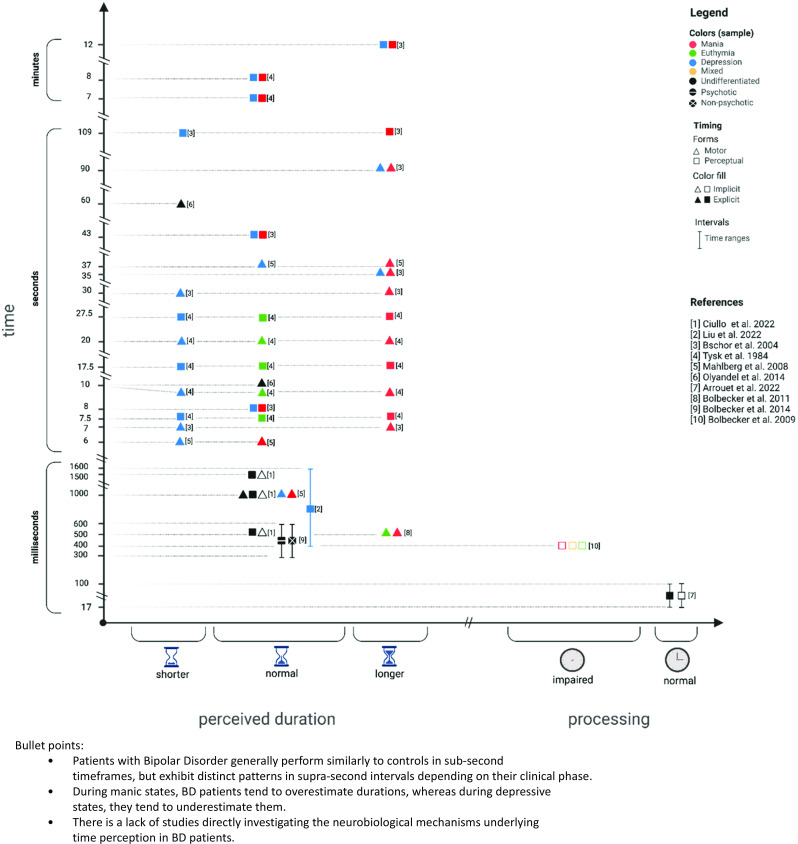

Summation

- Patients with bipolar disorder generally perform similarly to controls in sub-second timeframes, but exhibit distinct patterns in supra-second intervals depending on their clinical phase.
- During manic states, BD patients tend to overestimate durations, whereas during depressive states, they tend to underestimate them.
- There is a lack of studies directly investigating the neurobiological mechanisms underlying time perception in BD patients.

Considerations

- The review is limited by the small and heterogeneous sample size, potentially introducing confounds.
- Many studies did not differentiate patients based on their clinical states and type of bipolar disorder, particularly in sub-second timeframes.
- There is a significant lack of robust longitudinal research using experimental methodologies to assess temporal perception shifts across individual clinical phases.

## Introduction

Bipolar disorder (BD) is a prevalent condition that affects 2–5% of the global population (Merikangas *et al*., 2007). It is a chronic and hereditary illness characterised by periodic episodes of mania or hypomania and depression, which may each present with or without mixed features (McIntyre *et al*., 2020a). The disorder manifests through dramatic shifts in mood, significant fluctuations in energy, disruptions in biological clocks, and irregularities in sleep and social rhythms. In addition, individuals with BD often demonstrate a reduced adaptability to stress and a heightened sensitivity to environmental triggers (Pagani *et al*., 2016; McIntyre *et al*., 2020b). This disorder exhibits significant heterogeneity during clinical presentation, illness courses, treatment response, and functional outcome (McIntyre *et al*., 2022). The bipolar disorder spectrum is composed of BD type I, characterised by the occurrence of a syndromic manic episode, and BD type II, which is distinguished by the presence of a syndromic hypomanic episode and a major depressive episode. Although progress has been made to understand the pathophysiology of BD, the mechanisms underlying the disorder remain largely obscure (McIntyre *et al*., 2020b). Notably, emerging evidence suggests that disturbances in time perception may play a central role in the diverse symptomatology of BD (Northoff *et al*., 2018). To shed light on this topic, this work aims to conduct a systematic review of the experimental studies investigating time perception in patients with a diagnosis of BD. By synthesising the existing literature, this review provides a comprehensive understanding of this aspect and will potentially inspire further research in this promising direction.

### Psychopathology of time

Because of the profound connection between the human sense of being, self-awareness, and the experience of time, the study of time has long been a central issue in both phenomenological psychopathology and philosophy. In recent decades, a growing body of evidence suggests that the clinical symptoms of psychiatric disorders can be understood as the manifestations of underlying disturbances within the fundamental elements of consciousness, including the perception of time (Fuchs, 2013; Northoff, 2013; Arstila and Lloyd, 2014; Tewes and Stanghellini, 2020). The initial investigations on time perception for people suffering from mental disorders focused on schizophrenia (De La Garza and Worchel, 1956, Goldstone and Lhamon, 1956; Rabin, 1957, Ehrentheil and Jenney, 1960, Pearl and Berg, 1963; Simmel, 1963). They revealed a widespread alteration of time perception across short and long temporal intervals, with a dependence on arousal levels and rewards (for a review, see Ciullo *et al*., 2016; Thoenes and Oberfeld, 2017; Amadeo *et al*., 2022). Following these early studies on schizophrenia, a growing body of literature has explored the time domain in other neuropsychiatric disorders, including major depressive disorder (Thones and Oberfeld, 2015; Stanghellini *et al*., 2017), autism spectrum disorder (Vogel *et al*., 2018; Casassus *et al*., 2019), eating disorders (Stanghellini *et al*., 2017), and BD (see Results). Thus, numerous examples of abnormal time experiences are found in the classic phenomenological investigations of psychiatric disorders (Mezey and Cohen, 1961; Lehmann, 1967; Kitamura and Kumar, 1983; Tysk, 1984). This suggests that the disintegration of time has profound psychopathological effects on how one perceives oneself, the sensory world, and interactions with others (Kent and Wittmann, 2021). About BD, literature highlights distorted subjective temporal experiences, such as perceiving the passage of time as faster during mania and slower during depression (Bschor *et al*., 2004; Moskalewicz and Schwartz, 2020), and a past-focused time perspective during depression and a present/future-focused time perspective during mania (Ghaemi, 2007; Vogeley and Kupke, 2007; Gruber *et al*., 2012; Stanghellini *et al*., 2016; Stanghellini *et al*., 2017).

Northoff’s Spatiotemporal Psychopathology model (STPP) proposed that the experience of individual time, as well as space, is directly related to the neuronal topography and dynamics of the brain’s spontaneous activity (Northoff *et al*., 2018; Northoff *et al*., 2023; Northoff and Hirjak, 2023). That is, the spatiotemporal configurations of the brain contribute to spatiotemporal changes on the psychological level. In turn, this leads to different kinds of psychopathological symptoms, including affective, perceptual, cognitive, and motor symptoms. Regarding BD, the model proposed that its typical oscillations in symptomatology are underlined by cyclic desynchronisation between self-time and world-time, which potentially corresponds to changes in neuronal variability within the somatomotor and sensory resting state networks (Martino *et al*., 2016; Northoff *et al*., 2018). By bridging the phenomenological level with neurobiology, this new perspective implies not only a meticulous analysis of the existing findings on time alterations in BD but also a shift from the phenomenological qualitative investigation of subjective time experience to quantifying its subjective features through experimental paradigms and objective measurements. Despite the existence of only limited research, this review will go beyond the phenomenological level and explore the results of quantitative studies investigating time perception in BD patients and consider psychopathological and cognitive correlates.

By examining relevant research about time perception (Grondin, 2008; Vatakis *et al*., 2018), findings will be discussed considering whether i) explicit or implicit time perception was investigated, ii ) the temporal intervals involved were sub-second or supra-second, and iii) a perceptual or motor timing paradigm was used. Indeed, there is a typical distinction in time literature between explicit and implicit time perception (Coull and Nobre, 2008; Piras and Coull, 2011). Explicit time perception involves making explicit judgements about the temporal properties of external stimuli. For example, this includes comparing the duration of different stimuli, evaluating the temporal order of their appearance, or determining whether they are presented simultaneously or not. In contrast, implicit perception occurs automatically whenever we rely on intrinsic temporal information to solve other perceptual tasks. Indeed, some tasks involving perceptual judgements about other stimulus features implicitly require to use the temporal information intrinsic to stimulus presentation. An example is when individuals are asked to evaluate whether moving stimuli will collide: even though the task does not explicitly ask to evaluate time, temporal information, such as the speed of the sensory stimuli, is essential for predicting their eventual locations. Moreover, researchers have suggested that different mechanisms are involved depending on the temporal scale of external stimuli, specifically whether the intervals to be processed are above or below one second (e.g. evaluating the durations of stimuli lasting 10–200 ms or 10–200 s). Sub-second intervals reflect lower-level automatic timing processing, whereas supra-second intervals reflect higher-level cognitive timing processing (Mioni *et al*., 2020; Li *et al*., 2022). In addition, traditionally, the literature distinguishes between perceptual and motor timing (e.g. Keele *et al*., 1985; Jones and Jahanshahi, 2014). In perceptual timing, judgements of time are given through perceptual decisions. For instance, individuals typically state whether one temporal interval is shorter or longer than another, evaluate which stimulus appears first, or detect when the next stimulus will appear. In contrast, in motor timing, judgements of time are expressed through a motor response. Classic examples include production and reproduction tasks, where individuals are asked to produce or reproduce a duration using a sustained, delayed, or periodic motor act. For instance, participants may be asked to press a button for 1 s (production task), to press a button for the same duration as a sound presented through headphones, or reproduce a presented rhythm by tapping (i.e. reproduction tasks).

## Literature search and study selection

### Eligibility criteria

**Figure 1.**
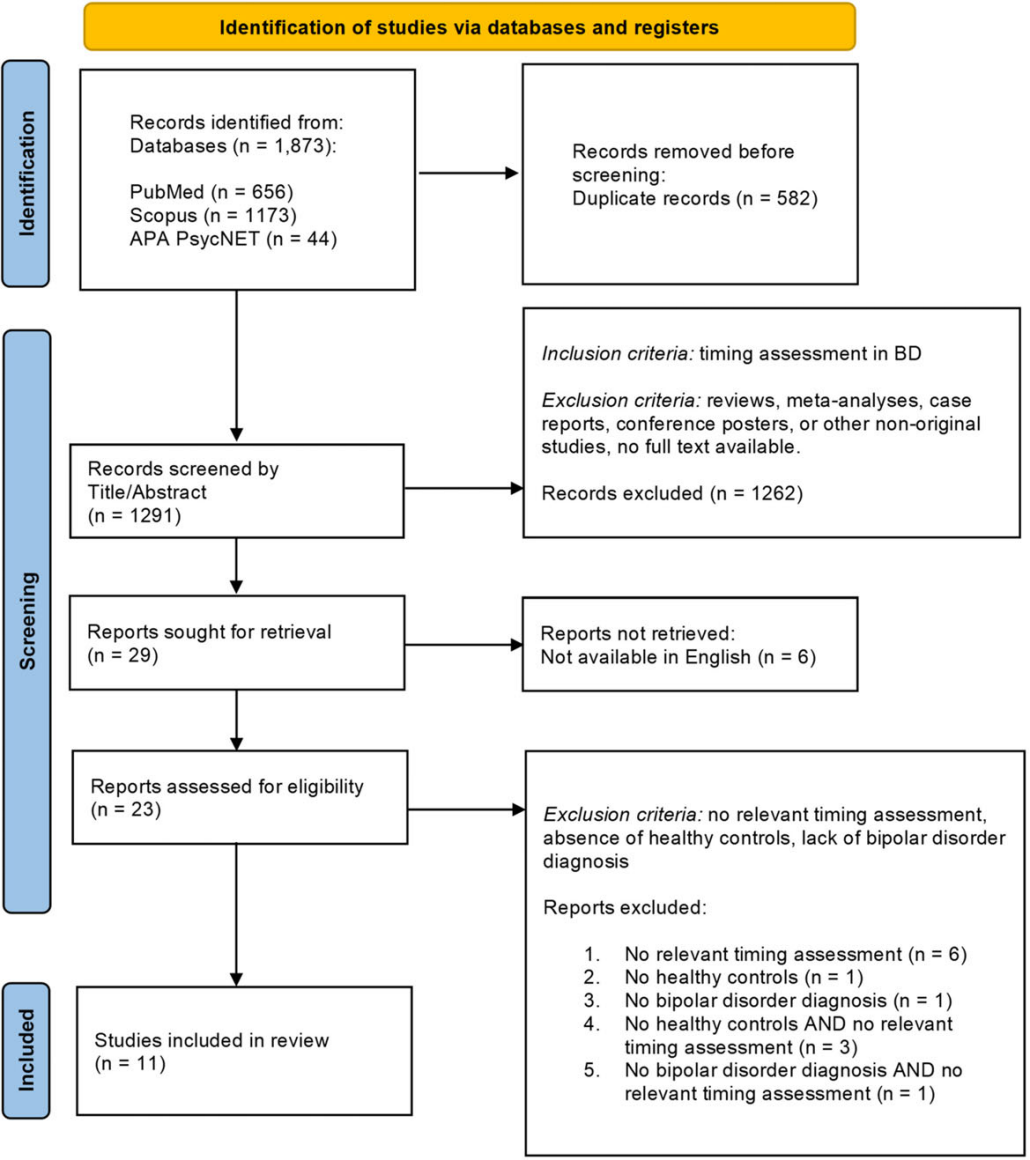
PRISMA flowchart of the literature search and study selection process.

The current study followed the Preferred Reporting Items for Systematic Reviews and Meta-analyses (PRISMA) recommendations (Page *et al*., 2021) to ensure a comprehensive inclusion of relevant articles on time perception in BD. We included studies published in English in peer-reviewed journals, without setting restrictions on the publication date. Since the goal of the systematic review is to illustrate quantitative results about time perception skills in BD, we have selected only studies encompassing an experimental paradigm to objectively measure the capacity of a subject to judge time. As such, the inclusion criteria were as follows: i) the presence of a bipolar and related disorders diagnosis based on the previous and latest ICD (World Health Organization, 2022) and DSM (Association, 2022) criteria; and ii) the use of experimental tasks to quantitatively investigate time perception as described in the manual “Timing and Time Perception: Procedures, Measures, and Applications” (Vatakis *et al*., 2018). The exclusion criteria were as follows: i) an absence of healthy controls; ii) a lack of a bipolar and related disorders diagnosis based on the previous and latest ICD (World Health Organization, 2022) and DSM (Association, 2022) criteria; iii) the lack of a timing assessment task as defined by “Timing and Time Perception: Procedures, Measures, and Applications” (Vatakis *et al*., 2018); iv) reviews, meta-analyses, case reports, conference posters, and other non-original studies; and v) studies in which the full text is not available. See Fig. 1 for the PRISMA flowchart and Supplementary Materials for the PRISMA check-list.

### Data sources

An extensive literature review was conducted by consulting PubMed, APA PsycNET, and Scopus. There were no limitations on the publication year, and the review covered the period up to July 2023. The search string used was (“bipolar disorder” OR “bipolar illness” OR “bipolar disease” OR “manic-depress*” OR “manic depress*” OR mania OR manic OR hypomanic* OR “mixed episode*” OR “mixed state*” OR “bipolar depress*” OR “bipolar spectrum” OR cyclothymic* OR “affective disorders” OR “mood disorders”) AND (“time perception” OR “temporal perception” OR “time estimation” OR “time judgment*” OR “subjective time” OR “subjective temporal” OR “temporal processing” OR “temporal binding” OR “timing” OR “tempo” OR “temporality” OR “duration judgment” OR “duration perception” OR “passage of time” OR “time perspective” OR “temporal perspective” OR “temporal order” OR “time sensitivity” OR “internal clock”).

### Data extraction

The following variables were extracted: i) first author; ii) year of publication; iii) title; iv) PMID/doi; v) investigated time range; vi) diagnosis; vii) sample size; viii) type of controls; ix) number of controls; x) female percentage (sample/controls); xi) mean age (sample/controls); xii) sample characteristics (inpatients or outpatients); xiii) clinical characteristics (clinical phase, ongoing pharmacological treatment); xiv) implicit or explicit timing task; xv) perceptual or motor timing task; xvi) modality of time assessment; xvii) index used for time assessment; xviii) cognitive, psychopathological, and neurobiological assessments (if present) along with their correlation with timing task performance; and xix) main findings of the studies.

### Study selection

The review process was facilitated by utilising Rayyan (Ouzzani *et al*., 2016), an open-source software used to manage reviews. The authors who conducted the literature screening independently evaluated the titles and abstracts of all the search results (AE, YM, MG). Any discrepancies between the researchers were resolved through discussion (AE, MBA, and MG). Initially, the articles were screened based on their titles and abstracts. Subsequently, there was a full-text assessment. Duplicate articles, review articles, and those not meeting the search criteria were excluded from further analysis.

### Assessment of methodological quality

The Appraisal Tool for Cross-Sectional Studies (AXIS) Scale (Downes *et al*., 2016) was used to assess the methodological quality of the studies included in the review. This choice was justified as the AXIS Scale is well-suited for evaluating the quality and risk of bias in cross-sectional research, ensuring an accurate interpretation of findings. It provides a structured approach to critically appraise studies based on several key criteria, including the clarity of aims, appropriateness of study design, justification of sample size, and the robustness of outcome measurement and statistical analysis.

## Results

### Literature search

The search yielded 1873 abstracts. After duplicates were removed, we screened 1291 records by title/abstract. Of these, 29 were relevant to our topic and included an experimental task to quantitatively assess time perception. Of these, 6 documents were excluded as they were not written in English. A full-text screening was performed on the remaining 23. Of these, 11 articles that were published between 1984 and 2022 were deemed eligible for inclusion. The process for selecting relevant articles is detailed in the Supplementary Materials (Table S1). The PRISMA flowchart, with details on screening and reasons for exclusion, is reported in Fig. 1. Results of the AXIS Scales for the assessment of methodological quality are reported in Supplementary Materials (Table S2). Studies show an overall high quality and low risk of bias, except for those by Tysk (1984) and Ryu *et al.* (2015) which have moderate quality and risk of bias.

### Sample size and clinical characteristics

Table 1 reports details about sample size and clinical characteristics. The review comprised 11 case–control studies involving 455 individuals with bipolar disorder as well as 545 healthy controls. While three studies conducted cognitive assessments (Bschor *et al*., 2004; Bolbecker *et al*., 2014; Ciullo *et al*., 2022), the other nine focused on psychopathological assessments (Bschor *et al*., 2004; Mahlberg *et al*., 2008; Bolbecker *et al*., 2009; Bolbecker *et al*., 2011; Bolbecker *et al*., 2014; Ryu *et al*., 2015; Arrouet *et al*., 2022; Ciullo *et al*., 2022; Liu *et al*., 2022); no study investigated neurobiological aspects. Regarding the BD patients’ clinical phases, the studies included 105 individuals during euthymia, 121 subjects in manic or hypomanic states, 108 individuals in depressed states, and 8 individuals in mixed states. Notably, two studies did not specify the clinical phase of the BD patients (Bolbecker *et al*., 2014; Oyanadel and Buela-Casal, 2014). Most studies did not mention comorbidities in their BD samples, with the exception of Ciullo *et al*. (2022), where three subjects had comorbid borderline personality disorder, and Liu *et al*. (2022); Arrouet *et al*. (2022), and Ryu *et al.* (2015) who explicitly reported no comorbid conditions. The other studies, such as Bolbecker *et al*. (2011, 2014, 2009), Mahlberg *et al*. (2008), Bschor *et al*. (2004), and Tysk (1984), either did not assess or did not provide information about comorbidities. In terms of the BD samples, one study included 50% of patients with psychotic features (Bolbecker *et al*., 2014). Three studies included a sample of SZ subjects (Bolbecker *et al*., 2014; Oyanadel and Buela-Casal, 2014; Arrouet *et al*., 2022). Moreover, three others included a sample of subjects with depressive symptoms (Tysk, 1984; Oyanadel and Buela-Casal, 2014; Liu *et al*., 2022). Further information is reported in Table 1.

**Table 1.** Sample characteristics of selected studies

Article	BD sample size	Clinical details and comorbidity	Control sample size	Patient age (mean years old ± sd)	Control age (mean years old ± sd)	Inpatients/ outpatients	Treatment
Ciullo *et al*., 2022	30 (50%F) (20 type I, 10 type II)	16 euthymic; 10 depressed; 4 hypoManic Comorbidities: 3 subjects with borderline personality disorder	30 (50%F)	42.27 ± 12.31	42.73 ± 12.87	Outpatients	Mood stabilisers and/or antidepressants; 20 CPZE
Liu *et al*., 2022	30 (66.7% F)	30 depressed Comorbidities: absent	30 (43.3% F)	26.09 ± 10.27	27.13 ± 4.66	Inpatients	N/A
Arrouet *et al*., 2022	20 (75.70 % F)	20 euthymic Comorbidities: absent	30 (50% F)	37.55 ± 10.67	37.73 ± 9.96	N/A	10 atypical antipsychotic, 1 antiparkinsonian, 13 anticonvulsant, 4 antidepressant, 12 lithium
Ryu *et al.,* 2015	46 (47.8% F)	22 manic (50.0% F); 24 euthymic (45.8% F) Comorbidities: absent	24 (58.3% F)	Manic BD: 30.27 ± 6.91; Euthymic BD: 30.75 ± 5.60	27.75 ± 2.74	Inpatients	CPZE; Manic: 10 lithium/divalproex, Euthymic: 10 lithium/divalproex
Bolbecker *et al*., 2014	71 (57.7% F)	34 with psychotic features (58.8% F); 37 without psychotic features (56.8% F) Comorbidities:N/A	73 (60.3% F)	N/A	N/A	Outpatients	BD with psychotic features: 5 unmedicated; 25 atypical antipsychotics; 1 typical antipsychotics; 10 anticonvulsants; 6 antidepressants; 0 anticholinergic; BD without psychotic features: 7 unmedicated; 23 atypical antipsychotics; 3 typical antipsychotics; 12 anticonvulsants; 9 antidepressants; 0 anticholinergic
Oyanadel *et al*., 2014	42 (81% F)	N/A Comorbidities:N/A	167	40.26 ± 11.13	37.38 ± 13.05	Outpatients	N/A
Bolbecker *et al*., 2011	42 (59.5% F)	17 manic; 25 euthymic Comorbidities:N/A	42 (52.4% F)	41.0 ± 11.5	40.9 ± 11.5	N/A	8 unmedicated, 25 antipsychotics, 25 mood stabilisers, 9 antidepressants, 1 anticholinergic
Bolbecker *et al*., 2009	28 (57.1% F)	9 manic; 8 mixed; 11 euthymic Comorbidities:N/A	28 (50.0% F)	41.39 ± 11.89	41.79 ± 12.02	N/A	7 unmedicated, 10 atypical antipsychotics, 1 typical antipsychotic, 11 mood stabilisers, 6 SSRI/tricyclic antidepressants
Mahlberg *et al*., 2008	56 (67.9% F)	28 manic (57.1% F); 28 depressed (78.6% F) Comorbidities:N/A	30 (53.3% F)	Manic BD: 46.4 ± 13.4; Depressed BD: 51.7 ± 15.7	46.1 ± 18	Inpatients	Lithium and/or other mood stabilisers; antidepressants
Bschor *et al*., 2004	62 (69.4% F)	30 manic (56.7% F), 32 depressed (81.3% F) Comorbidities:N/A	31 (58.1% F)	Manic BD: 47.0 ± 13.2; Depressed BD: 52.8 ± 15.3	45.7 ± 17.8	Inpatients	Manic: 25 lithium, 4 carbamazepine, 8 valproate, 0 tricyclic antidepressants, 1 SSRI, 0 venlafaxine, 0 mirtazapine, 16 benzodiazepines, 14 neuroleptics; Depressed: 9 lithium, 3 carbamazepine, 1 valproate, 12 tricyclic antidepressants, 7 SSRI, 4 venlafaxine, 5 mirtazapine, 8 benzodiazepines, 6 neuroleptics
Tysk, 1984	28	11 manic or hypomanic; 8 depressed; 9 in remission Comorbidities:N/A	60	N/A	N/A	Inpatients	Psychotropic drugs

### Features of time perception

Based on the literature review, we found that 10 studies involved explicit time perception (Tysk, 1984; Bschor *et al*., 2004; Mahlberg *et al*., 2008; Bolbecker *et al*., 2011; Bolbecker *et al*., 2014; Oyanadel and Buela-Casal, 2014; Ryu *et al*., 2015; Arrouet *et al*., 2022; Ciullo *et al*., 2022; Liu *et al*., 2022), three studies involved implicit time perception (Bolbecker *et al*., 2009; Arrouet *et al*., 2022; Ciullo *et al*., 2022), six studies investigated sub-second temporal intervals (Bolbecker *et al*., 2009; Bolbecker *et al*., 2011; Bolbecker *et al*., 2014; Arrouet *et al*., 2022; Ciullo *et al*., 2022; Liu *et al*., 2022), seven studies investigated supra-second temporal intervals (Tysk, 1984; Bschor *et al*., 2004; Mahlberg *et al*., 2008; Oyanadel and Buela-Casal, 2014; Ryu *et al*., 2015; Ciullo *et al*., 2022; Liu *et al*., 2022), eight studies involved perceptual timing (Tysk, 1984; Bschor *et al*., 2004; Bolbecker *et al*., 2009; Bolbecker *et al*., 2014; Ryu *et al*., 2015; Arrouet *et al*., 2022; Ciullo *et al*., 2022; Liu *et al*., 2022), and seven studies focused on motor timing (Tysk, 1984; Bschor *et al*., 2004; Mahlberg *et al*., 2008; Bolbecker *et al*., 2011; Oyanadel and Buela-Casal, 2014; Ryu *et al*., 2015; Ciullo *et al*., 2022). Table 2 summarizes the studies based on time perception features.

**Table 2.** Comparative studies on explicit and implicit timing tasks by temporal interval and response type

	Explicit		Implicit	
	Perceptual timing	Motor timing	Perceptual timing	Motor timing
Sub-second	Arrouet *et al*., 2022, Ciullo *et al*., 2022, Liu *et al*., 2022, Bolbecker *et al*., 2014	Bolbecker *et al*., 2011	Arrouet *et al*., 2022, Bolbecker *et al*., 2009	Ciullo *et al*., 2022
Supra-second	Ciullo *et al*., 2022, Liu *et al*., 2022, Ryu *et al*., 2015, Bschor *et al*., 2004, Tysk, 1984	Ciullo *et al*., 2022, Ryu *et al*., 2015, Oyanadel *et al*., 2014 Mahlberg *et al*., 2008, Bschor *et al*., 2004, Tysk, 1984		Ciullo *et al*., 2022

### Main findings

**Figure 2.**
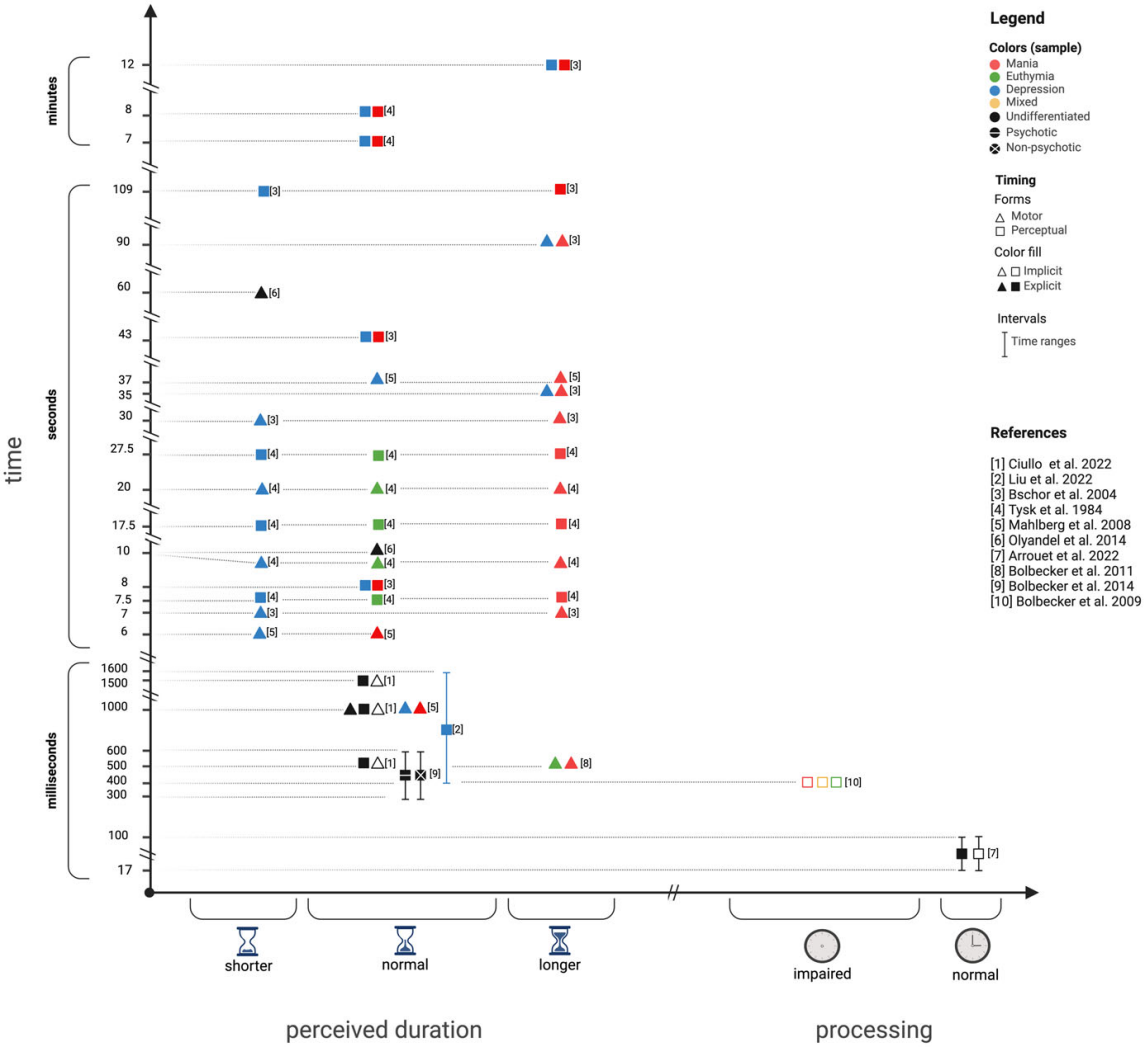
Results of each study are represented based on BD performance at specific temporal intervals. Left: perceived duration, right: time processing performance. *X*-axes: shorter = underestimation compared to controls, normal = estimation similar to controls, longer = overestimation compared to controls; impaired = worst performance compared to controls, normal = similar performance compared to controls. *Y*-axes: timeframes investigated. Colours: red = BD during manic state, green = BD during euthymic state, blue = BD during depressed state, black = BD with no other specifications, black with white line = BD with psychotic features, black with white cross = BD without psychotic features. Forms: triangle = motor timing, square = perceptual timing. Fill: filled = explicit time perception, empty = implicit time perception.

The main findings for each study are reported in Table 3. Table 4 divides the studies by experimental paradigm and interval range investigated, and Fig. 2 represents a graphical summary of their results.

**Table 3.** Assessment details and main findings of selected studies

Article	Temporal paradigm	Time range	Implicit/ explicit	Sub-second/ supra-second	Perceptual/ motor	Psychopathological assessment	Cognitive assessment	Main findings	Psychopathological Correlates	Cognitive Correlates
Ciullo *et al*., 2022	*Discrimination:* judge the relative duration of two consecutive stimuli; *Bisection:* determine whether the perceived duration of an empty interval was longer or shorter than 1 s; *Reproduction:* reproduce the perceived duration of 1 s; P*redictive timing*: detect as quickly as possible a stimulus during manipulation of temporal expectations	*Discrimination* *Bisection* *Reproduction* *Predictive timing:* 540 ms, 1080ms, 1620ms	Both	Both	Both	YMRS; HAM-D; PANSS; MMSE; CPZE; number of depressive, manic, and hypomanic episodes	DIR; TMT-A	*Discrimination:* BD performance similar to controls; *Bisection:* BD performance similar to controls; *Reproduction:* BD accuracy similar to controls but reduced precision; *Predictive timing*: BD performance similar to controls	Number of previous depressive episodes was a predictor of precision at temporal *Reproduction*	Accuracy at DIR was a predictor of precision at temporal reproduction
Liu *et al*., 2022	*Bisection:* rate as “Short” or “Long” the length of a given temporal interval	400–1600ms	Explicit	Both	Perceptual	HAM-D	None	BD performance similar to controls	No significant results considering the psychopathological assessment	N/A
Arrouet *et al*., 2022	*Temporal order judgement:* determine the order of appearance of two targets	0 ms, 17 ms, 100ms	Both	Sub-second	Perceptual	CPZE	None	*Explicit measures:* BD patients performed similar to controls in both 17 ms and 100 ms tasks *Implicit measures*: no difference between BD patients and controls	No correlation between performance and CPZE	N/A
Ryu *et al*., 2015	*Estimation:* identify the duration of presentation of affective pictures; *Reproduction*: reproduce the duration of affective pictures	2s, 4s, 6s	Explicit	Supra-second	Both	YMRS; MADRS; BPRS; CGI; CPZE	None	*Estimation* euthymic BD performance similar to controls, while no effect of positive low-arousal pictures for manic BD; *Reproduction*: euthymic BD performance similar to controls; reversed effect of all emotional stimuli for manic BD	Euthymic patients were similar to controls, while manic patients showed a different pattern; significant correlations emerged only for the manic group between performance at *estimation* task and the manic and illness symptom severity, as measured by the YMRS and the CGI.	N/A
Bolbecker *et al*., 2014	*Bisection:* rate as “Short” or “Long” the length of a stimulus	300–600ms	Explicit	Sub-second	Perceptual	YMRS; MADRS	WASI-IQ	BD accuracy similar to controls but reduced precision	No differences between BD with and without psychotic features; no significant results considering the psychopathological assessment	No significant results
Oyanadel *et al*., 2014	*Production*: produce empty intervals	10s, 60s	Explicit	Supra-second	Motor	None	None	BD performance similar to control for 10s but underestimation of 60s	N/A	N/A
Bolbecker *et al*., 2011	*Production:* paced finger-tapping task with i) dominant index finger and ii) alternating thumbs	Inter tap interval of 500ms	Explicit	Sub-second	Motor	YMRS; MADRS; drugs	None	BD tapped at a faster rate than controls, specifically when using alternating thumbs, and showed an overall greater timing variability	No significant differences between manic and euthymic patients; no significant results considering the psychopathological assessment	N/A
Bolbecker *et al*., 2009	*Eyeblink conditioning task*: presence and temporal latency of eyeblink conditioning responses to auditory stimuli	Length of conditioned stimulus 400ms	Implicit	Sub-second	Perceptual	YMRS; MADRS; drugs	None	BD reported lower percentage and faster latencies of conditioned responses compared to controls	The timing deficit was predominantly observed in patients with a mixed episode; no significant results considering the psychopathological assessment	N/A
Mahlberg *et al*., 2008	*Reproduction:* press a key to reproduce durations	1s, 6s, 37s	Explicit	Supra-second	Motor	HDRS; BRMAS	None	Manic and Depressed BD similar to control for 1s; Manic BD accurately reproduced 6s and under-reproduced 37s; depressed BD over-reproduced 6s and accurately reproduced 37s.	Different performance between manic and depressed BD for 6s and 37s; no significant results considering the psychopathological assessment	N/A
Bschor *et al*., 2004	*Estimation:* evaluate the duration of stimuli; *Production*: produce by pressing a key some durations	*Estimation* 43s, 109s, 8s 12m40s; *Production*: 7s, 35s, 90s	Explicit	Supra-second	Both	HDRS; BRMAS; duration of disorder, number of prior episodes, duration of the current episode	TMT-A	*Estimation:* manic and depressed BD similar performance to control for 8s e 43s, manic BD reported longer duration and depressed BD shorter duration for 109s, manic BD reported longer durations for 12min; *Production:* manic BD produced shorter duration and depressed BD produced longer duration for 7s, manic and depressed BD produced shorter durations for 35s and 90s	Different performance between manic and depressed patients; poorer performance in both tasks was linked with the number of previous episodes for depressed BD	Worst performance at TMT-A was associated with an overestimation in time *estimation* in manic BD
Tysk, 1984	*Estimation:* judge durations of stimuli; *Metronome adjustment* adjustment: set the metronome to 1beat/s *Production*: produce temporal interval with a stopwatch	*Estimation* 7.5s, 17.5s, 27.5s 7/8min; *Metronome adjustment*: tempo range 40 to 208 beats/min to adjust to 1 beat/s P*roduction*: 10s, 20s, 30s	Explicit	Supra-second	Both	None	None	*Estimation* depressed BD tended to underestimate and manic BD tended to overestimate 7.5s, 17.5s, and27.5s, depressed and manic BD estimated correctly 7/8min; *Metronome adjustment:* expressed BD tended to underestimate and manic BD tended to overestimate the rhythm; *Production*: depressed BD underestimated and manic BD tended to overestimated 10s, 20s and 30s. Performance of BD in remission similar to controls for all tasks	Depressed BD tended to underestimate time, manic BD tended to overestimate time, BD in remission performed similar to controls	N/A

BD, bipolar disorder; YMRS, Young Mania Rating Scale (Young, *et al*., 1978); HAM-D, Hamilton Depression Rating Scale (Hamilton, 1960); CPZE, chlorpromazine equivalents; PANSS, Positive and Negative Syndrome Scale (Kay, *et al*., 1987); BPRS, the Brief Psychiatric Rating Scale (Pull & Overall, 1977); MADRS, the Montgomery–Åsberg Depression Scale (Montgomery & Åsberg, 1979); CGI, Clinical Global Impression; BRMS, Bech–Rafaelsen Mania Scale (Bech, *et al*., 1978).

**Table 4.** List of studies by experimental paradigm and temporal interval range

	Sub-second <1 s	Supra-second short 1s–2s	Supra-second medium 2s–5 min	Supra-second long >5 min
Estimation		Ryu *et al*., 2015	Ryu *et al*., 2015, Bschor *et al*., 2004, Tysk, 1984	Bschor *et al*., 2004
Discrimination	Ciullo *et al*., 2022, Liu *et al*., 2022, Bolbecker *et al*., 2014	Ciullo *et al*., 2022, Liu *et al*., 2022		
Metronome adjustment		Tysk, 1984		
Temporal order judgement	Arrouet *et al*., 2022			
Predictive timing	Ciullo *et al*., 2022	Ciullo *et al*., 2022		
Production	Bolbecker *et al*., 2011		Oyanadel *et al*., 2014, Bschor *et al*., 2004, Tysk, 1984	Tysk, 1984
Reproduction	Ciullo *et al*., 2022	Ciullo *et al*., 2022, Ryu *et al*., 2015, Mahlberg *et al*., 2008	Ryu *et al*., 2015, Mahlberg *et al*., 2008	
Reflex	Bolbecker *et al*., 2009			

#### Explicit timing

*Brief overview of each study.*

- Ciullo *et al.* (2022) investigated temporal perception by administering various explicit motor and perceptual paradigms to BD patients (BD type I, *n* = 20; BD type II, *n* = 10) involving both *sub-second* and *supra-second* stimuli. Specifically, they conducted the following tasks: i) *duration discrimination task*: participants were tasked with judging the relative duration (shorter, equal to, or longer) of two consecutive visual dots that lasted either 540 ms, 1080 ms, or 1620 ms; ii) *temporal bisection task*: participants determined whether the perceived duration of an empty interval was longer or shorter than their internal representation of 1s; and iii) *temporal reproduction task*: participants were required to reproduce, using a motor response (finger tapping), the perceived duration of an empty 1s interval. Compared to control participants (*n* = 30), individuals with BD showed no significant differences in measures of duration discrimination and temporal bisection. However, while their accuracy (i.e. mean reaction times (RT)) was comparable, BD participants exhibited reduced precision (i.e. greater standard deviation of RTs) in reproducing a one-second duration relative to the control group.

- Liu *et al.* (2022) performed an explicit task of perception of time using both *sub-second* and *supra-second* stimuli in BD individuals with a current major depressive episode (*n* = 30), healthy controls (*n* = 30), and individuals with a diagnosis of major depressive disorder (*n* = 30). The task consisted of a *temporal bisection task* divided into two phases. During the training phase, participants were required to memorise two temporal intervals showed through visual stimuli (short: 400 ms, long: 1600 ms). During the testing phase, participants were instructed to rate as “Short” or “Long” the length of a given temporal interval (ranging from 400 to 1600 ms). While the BD group showed a tendency to overestimate sub-second intervals and underestimate supra-second intervals, there were no significant differences in precision (i.e. difference limen and Weber ratio) and accuracy (i.e. point of subject equality (PSE)) compared to the control group.

- Arrouet *et al.* (2022) tested *sub-second* temporal skills of BD patients (*n* = 20), individuals with schizophrenia (*n* = 24), and healthy controls (*n* = 31) using a *temporal order judgement task*. The latter consisted of asking participants to determinate the order of presentation of two visual stimuli (stimulus onset asynchrony could be 17 ms or 100 ms). Differently from most other paradigms mentioned in this review, this kind of task examines a different domain of time, that is the processing of temporal structure of events. Interestingly, while patients with schizophrenia performed worse as compared to controls, patients with BD showed a performance in-between that of controls and patients with schizophrenia, with no differences compared to either groups.

- Ryu *et al.* (2015) examined temporal estimation and reproduction skills in the *supra-second* domain in BD patients, splitting the sample based on the clinical episode; a manic state (*n* = 22) and euthymic state (*n* = 24). Since authors were interested in the relationship between emotions and temporal perception, they choose as stimuli standardised affective pictures with different valence and level of arousal. In the *temporal estimation task*, participants identify the duration of presentation of each picture (2s, 4s, or 6s) on a range from 1 to 10 s in a visual analogue scale. In the *temporal reproduction task*, they were asked to reproduce the duration by pushing a button. While for both tasks the overall performance of euthymic subjects and healthy individuals similarly varied based on valence and level of arousal, this was not the case for the manic group. Indeed, both euthymic patients and healthy controls consistently overestimated positive low-arousal and negative high-arousal pictures and consistently underestimated positive high-arousal and negative low-arousal pictures. Instead, for manic patients there was no effect of positive low-arousal pictures during the estimation task, and there was a reversed effect of all emotional stimuli during the reproduction task. Since supra-second durations require more attentional resources than sub-seconds ones (Droit-Volet *et al*., 2013), and based on previous literature which relates attentional components to emotional processing (e.g. Angrilli *et al*., 1997; Strakowski *et al*., 2011), the authors suggested that impaired attentional processes may affect temporal judgements of emotional stimuli during manic phase.

- Bolbecker *et al.* (2014) investigated explicit *duration* for *sub-second* intervals in subjects with non-psychotic BD (*n* = 37), BD with psychotic features (*n* = 34), schizophrenia (*n* = 66), and schizoaffective disorder (*n* = 31), as well as in a healthy control group (*n* = 73). The task was a temporal bisection that required participants to rate as “Short” of “Long” auditory durations of tones ranging from 300 to 600 ms. The testing phase was preceded by a training and a practice session where participants learned the anchor durations (i.e. 300 ms and 600 ms). While accuracy (i.e. bisection point) was consistent across all groups, all BD patients and individuals with schizophrenia exhibited significantly increased variability (i.e. worst precision as revealed by higher difference limen and Weber ratio).

- Oyanadel and Buela-Casal (2014) investigated explicit *supra-second* perception of time with a *time estimation task* (time interval and analyses not specified) and a *time production task* (10s, and 60s) in BD subjects (*n* = 42), patients with major depressive disorder (*n* = 70), schizophrenia (*n* = 30), cluster B personality disorder (*n* = 25), and healthy participants (*n* = 167). While groups did not significantly differ from each other in the estimation task (details of results not provided) and production of 10s interval, patients with BD and major depressive disorder underestimated time when producing intervals of 60s.

- Bolbecker *et al*. (2011) evaluated BD patients (*n* = 42) in a manic episode (*n* = 17) or euthymia (*n* = 25), and healthy controls (*n* = 42), using a *production timing task* involving *sub-second* stimuli. This involved a finger-tapping task where participants were directed to tap their index finger or alternate their thumbs in sync with a paced auditory stimulus (synchronization phase with a 500 ms inter-tap interval). Subsequently, they were instructed to continue tapping at the same rhythm even after the auditory stimulus ceased (continuation phase).The BD group tapped at a faster rate than controls, specifically when using alternating thumbs, and showed an overall greater timing variability (i.e. higher standard deviation of inter-tap interval). When analysing mood states within the BD group separately, no significant differences between manic and euthymic patients emerged.

- Mahlberg *et al.* (2008) conducted a *temporal reproduction task* involving *supra-second* stimuli with BD patients, either in acute mania (*n* = 28) or acute depression (*n* = 28), and with a control group of healthy individuals (*n* = 30). Participants were asked to press a key to reproduce stimuli of 1s, 6s or 37s. Although no differences emerged for the very short temporal span (i.e. 1s), manic BD patients generally perceived durations as shorter when compared to their depressed counterparts. Yet, the pattern of response diverged between short and long temporal spans. Manic patients accurately reproduced the short interval (6s), but under-reproduced the long interval (37s). Conversely, depressed patients reproduced the long interval accurately, but over-reproduced the short interval. Although it can sound counterintuitive, it is important to keep in mind that, in motor timing task, to produce/reproduce a shorter time span than demanded is considered overestimation, and to produce/reproduce a longer time span than demanded is defined as underestimation (Bindra and Waksberg, 1956).

- Bschor *et al.* (2004) investigated the *temporal estimation* and *temporal production* of *supra-second* stimuli in BD patients with a manic episode (*n* = 30), in BD patients with a depressive episode (*n* = 32), and controls (*n* = 31). After a training session, they were asked to evaluate the duration of visual stimuli lasting 8 s, 43 s, and 109 s, and to produce by pressing a key durations as long as 7s, 35s, and 90s. They were also asked to verbally report the duration of a short movie (12 min and 40s).

The results indicated that all participants performed similarly in the estimation task for the shorter temporal intervals (i.e. 8 s and 43 s). However, for the longer interval (i.e. 109 s), there were differences among BD groups: those in a manic episode reported longer durations, while those in a depressive episode reported shorter durations. When judging the duration of the short movie, depressed BD patients, and even more strongly manic BD patients, reported a significantly longer duration (i.e. 28 min±9 min) compared to controls.

In the production task, all BD patients produced shorter temporal intervals when evaluating longer temporal spans (i.e. 35 s and 90 s, but not 7 s). This effect was significantly more pronounced in BD patients with a manic episode compared to those with a depressive episode. Conversely, for the shorter intervals (i.e. 7 s), those with a manic episode produced significantly shorter durations, while those in a depressive episode longer durations. While not classified as an experimental paradigm according to the review criteria, it is worth mentioning that Bschor et al., explored also subjective time experience by asking participants to evaluate how slow or fast she or he experienced the flow of time on the day of the investigation. The control group conveyed a relatively balanced perception of time. In contrast, the depressed group reported a significant perception of time slowing down, while the manic group felt a marked acceleration.

- Tysk (1984) recruited healthy individuals (*n* = 60) alongside a substantial cohort of patient with affective disorders. This included BD subjects in (hypo)manic (*n* = 11 ), depressed (*n* = 8), or euthymic (*n* = 9) states; major depressive patients with (*n* = 9) and without (*n* = 9) melancholic features; and individuals with dysthymic disorder (*n* = 10). Different tasks were used to explicitly evaluate *supra-second* perception of time: *metronome adjustment* (tempo range 40 to 208 beats/minute and subjects had to set it to one beat per second), *verbal estimation* (7.5 s, 17.5 s, 27.5 s and 7/8 min), and *motor reproduction* (10 s, 20 s, and 30 s). In all tasks, patients with BD during a depressive episode and melancholic subjects tended to under-estimate the short time intervals, while (hypo)manic individuals tended to count seconds too fast and to overestimate time. For the longer interval verbal estimation (i.e. 7/8 min), both BD patients during manic and depressive states were comparable to the control group. Moreover, all other patient groups in the study, including BD patients in remission, generally showed results similar to those of the healthy participants.

*Summary of results for sub-second intervals.* In the domain of sub-second explicit timing, individuals with BD generally do not display significant alterations in perceptual timing compared to HC (Arrouet *et al*., 2022; Ciullo *et al*., 2022; Liu *et al*., 2022). In turn, similar results are observed in temporal discrimination (Ciullo *et al*., 2022), temporal bisection (Ciullo *et al*., 2022; Liu *et al*., 2022), and temporal order judgement (Arrouet *et al*., 2022). However, one study (Bolbecker *et al*., 2014) documented increased timing variability in a temporal bisection task in BD compared to HC; the researchers interpreted it as reduced precision in sub-second time estimation. Only one study investigated sub-second explicit motor timing; it determined that greater timing variability (higher standard deviation) and faster tapping rate characterised both euthymic and manic BD patients compared to HC (Bolbecker *et al*., 2011).

*Summary of results for supra-second intervals.* With regard to intervals from 1 to 2 s, no differences emerged in perceptual timing (Ciullo *et al*., 2022; Liu *et al*., 2022). However, the opposite results were documented for motor timing, with either similar performance between BD patients and controls (Mahlberg *et al*., 2008) or reduced precision in BD patients partially explained by the patients’ number of previous depressive episodes (Ciullo *et al*., 2022).

Supra-second timing for temporal intervals between 2 s and 5 min did instead reveal more consistent differences between groups. Within this temporal span, although some authors reported some similar results between individuals with BD and healthy controls in perceptual (for 8 s and 43 s: (Bschor *et al*., 2004)) and motor (for 10 s: (Oyanadel and Buela-Casal, 2014)) explicit timing, most studies noted a general divergence between mania and depression. Manic BD patients tend to overestimate temporal spans while depressed BD patients tend to underestimate them. Specifically, Tysk (1984) reported an overestimation during manic episodes and an underestimation in melancholic states in both the verbal estimation and motor production tasks encompassing temporal intervals between 7 and 30 s. According to Mahlberg *et al.* (2008), depressed subjects showed a tendency towards over-reproduction (i.e. underestimating) for 6 s temporal intervals, while manic patients showed a tendency towards under-reproduction (i.e. overestimating) for 37 s temporal intervals. Similarly, although all patients were found to overestimate 35 s and 90s intervals (production task), manic patients tended to overestimate while depressed patients tended to underestimate at durations of 7 s (production task) and 109 s (estimation task), according to the results by Bschor *et al*. (2004). In this case, the overestimation of time in the production task observed in manic BD patients correlated with impaired performance on the Trail Making Test part-A; in turn, the underestimation of depressed patients in both estimation and production tasks was predicted by the number of previous depressive episodes they had experienced. Oyanadel and Buela-Casal (2014) determined that BD patients underestimate 60 s during a production task, but they did not take into account illness phases. Within this temporal span, the findings by Ryu et al. (2015) demonstrated that emotional stimuli affected time perception differently in manic BD patients compared to euthymic patients and control individuals. However, these results are only partially related to the context of this review as they involved affective stimuli and investigated emotional effects on time perception rather than time perception. Only two studies considered longer time intervals (longer than 5 min). Tysk (1984) found no difference between manic and depressed BD patients and healthy controls in temporal estimation. Subsequently, Bschor *et al*. (2004) found that depressed and even more strongly manic individuals exhibited temporal overestimation.

#### Implicit timing

*Brief overview of each study.* Ciullo *et al*. (2022), in addition to the explicit paradigms, tested BD patients (BD type I, *n* =20; BD type II, *n* =10) with a *predictive timing task* involving both *sub-second* and *supra-second* stimuli. Participants were instructed to detect as quickly as possible visual targets, which appeared after one of three intervals (540 ms, 1080ms, or 1620ms). Temporal expectations were modulated using symbolic cues with an 80% validity rate. Patients did not differ from controls.

- As described in the Section 3.4.1.1, Arrouet *et al*. (2022) administered an explicit *temporal order judgement task* to BD patients (*n* = 20), individuals with schizophrenia (*n* = 24), and healthy controls (*n* = 31). However, authors conducted specific analyses based on trial n-1 which allowed to draw conclusions about the implicit mechanisms that optimise the processing of successive stimuli based on previous ones. Interestingly, the implicit order effects, which lead to improved performance, were preserved and similar across groups.

- Bolbecker *et al*. (2009) administered a *delay eyeblink conditioning task* using *sub-second* auditory stimuli to a group of BD patients (*n* = 28) during manic (*n* = 9), mixed (*n* = 8), and euthymic (*n* = 11) states, and a group of healthy participants (*n* = 28). The procedure involved assessing the acquisition and latency of conditioned eyeblink responses to an air puff (i.e. an unconditioned stimulus lasting 50 ms) that was paired with a tone (i.e. a conditioned stimulus lasting 400 ms). This kind of paradigm, similarly to Arrouet *et al.* (2022), investigates the temporal structure of how participants process external events. While all participants responded to the conditioning, the BD group exhibited a lower percentage of conditioned responses compared to controls. Upon grouping the BD group based on episodes, this trend was particularly pronounced for BD individuals in a mixed episode. More relevant to the topic of the review, BD patients differed from healthy participants regarding the latency of the conditioned response; specifically, they demonstrated faster latencies. This meant less adaptively timed responses for patients, as the timing of the conditioned response should gradually align more precisely with the onset of the unconditioned stimulus during the conditioning phase. Consequently, faster conditioned responses are less adaptive compared to those that occur later and closer to the onset of the unconditioned stimulus. Even in this case, BD patients during mixed episode performed worse than all other groups, failing to improve as the experiment progressed too. As for the peak latency of the unconditioned response, it decreased with experience similarly for all groups. Authors discussed these findings as evidence of poor temporal coordination of information processing, specifically in patients with mixed episode.

*Summary of results for sub-second and supra-second intervals.* Regarding implicit timing, one study reported a similar sensitivity between BD patients and controls to temporal expectations during a predictive timing task within the sub-second and supra-second range (Ciullo *et al*., 2022) and no alterations in sub-second implicit mechanisms underlying temporal processing were observed in another study using a temporal order judgement task (Arrouet *et al*., 2022). However, one study reported lower conditioned responses and faster latency in eye-blink reflex conditioned responses in the sub-second domain, especially for BD during mixed episodes (Bolbecker *et al*., 2009).

### Psychopathological correlates of time perception

The psychopathological assessment section mentioned only measures for which the association between the psychopathological indices and temporal performance had been tested.

Only one study compared BD type I and BD type II; it found similar results between the two groups (Ciullo *et al*., 2022). Although only a few studies analysed the performance of patients during different clinical states separately, those that did all highlight some significant differences between patients with depressive and manic episodes during supra-second tasks. Overall, manic patients tended to overestimate time, depressed patients tended to underestimate it, and patients in remission behaved similarly to the controls (Mahlberg *et al*., 2008; Tysk, 1984; Bschor *et al*., 2004). In terms of differences between euthymic and manic patients, Bolbecker et al. (2011) found no significant differences in sub-second finger-tapping performance, as well as Bolbecker et al. ( 2009), when investigating the presence and temporal latency of eyeblink conditioning responses to auditory stimuli during an implicit sub-second timing task (here, the timing deficit was predominantly observed in patients with mixed episodes).

When considering the number and/or duration of previous clinical episodes, these variables seem to be crucial only for depressive states. Indeed, Ciullo *et al.* (2022) found that the number of previous depressive episodes was a predictor of BD patients’ precision in the temporal reproduction of 1 s and Bschor *et al*. (2004) found that poorer performance at supra-second estimation and production tasks was linked with the number of previous episodes depressed participants had experienced.

Only one study (Bolbecker *et al*., 2014) directly addressed the impact of the presence of psychotic symptoms, but they found no differences between patients with and without psychotic symptoms during the sub-second bisection task. One other study indirectly investigated the role of psychotic features by testing patients with the PANSS; however, the scores at its sub-scales did not predict temporal performance (Ciullo *et al*., 2022).

A few studies evaluated the impact of medications and considered, for instance, whether patients were on antipsychotic medications. Interestingly, they all revealed no significant results (Bolbecker *et al*., 2009; Bolbecker *et al*., 2011; Ryu *et al*., 2015; Arrouet *et al*., 2022; Ciullo *et al*., 2022; Liu *et al*., 2022).

Some studies considered the association between temporal performance and psychopathological scales (i.e. the Young Mania Rating Scale (YMRS) (Young *et al*., 1978), the Hamilton Depression Rating Scale (HAM-D) (Hamilton, 1960), the Positive and Negative Syndrome Scale (PANSS) (Kay *et al*., 1987), the Brief Psychiatric Rating Scale (BPRS) (Pull and Overall, 1977), the Montgomery–Åsberg Depression Scale (MADRS) (Montgomery and Åsberg, 1979), the Clinical Global Impression (CGI), and the Bech–Rafaelsen Mania Scale (BRMS) (Bech *et al*., 1978)). Most of them failed to find a significant association between patients’ scores on the scales and their temporal skills (Ciullo *et al*., 2022) (Bolbecker *et al*., 2009; Bolbecker *et al*., 2014; Liu *et al*., 2022) (Mahlberg *et al*., 2008) (Bschor *et al*., 2004). Only one study found that the performance of manic patients at supra-second estimation task correlated with manic and illness symptom severity as measured by the YMRS and the CGI scales (Ryu *et al*., 2015). For details about each study, see Supplementary Materials.

### Cognitive correlates of time perception

Three of the selected studies investigated the cognitive correlates of time perception in BD. Two of them found significant results concerning the relationships between cognitive skills and temporal performance (Bschor *et al*., 2004; Ciullo *et al*., 2022) while the third one did not (Bolbecker *et al*., 2014). Specifically, the precision at temporal reproduction of BD patients was found to be influenced by working memory as measured by the Delayed Item Recognition task (DIR) (Ciullo *et al*., 2018b; Ciullo *et al*., 2022). In addition, executive and motor speed assessed with the Trail Making Test part-A (TMT-A) (Reitan, 1992) correlated with the overestimation of time in manic patients during an estimation task (Bschor *et al*., 2004). However, IQ information derived from the Wechsler Abbreviated Scale of Intelligence (WASI) (Wechsler, 1999) was not related to precision at a temporal bisection task (Bolbecker *et al*., 2014). For details about each study, see Supplementary Materials.

## Discussion

Time perception alterations are characteristic of severe mental disorders that exhibit diverse clinical and neurobiological manifestations (Giersch *et al*., 2016; Stanghellini *et al*., 2016; Tschacher *et al*., 2017). We aim to provide a thorough analysis of the literature concerning time perception in patients with BD. We included only studies that employed an experimental paradigm to objectively evaluate temporal performance. This allowed us to systematically categorise explicit/implicit and perceptual/motor timing alterations across sub-second/supra-second time intervals. To the best of our knowledge, this represents the first systematic review on the topic.

When addressing BD, the literature often refers to a subjective acceleration in manic individuals and a deceleration in patients with depression in the minute-to-day range. This conception is grounded in extensive psychopathological literature (Ghaemi, 2007; Vogeley and Kupke, 2007; Gruber *et al*., 2012; Stanghellini *et al*., 2016; Stanghellini *et al*., 2017). Although it seems to be a hypothesis with significant theoretical bases, and even if it sounds this way to clinicians in their daily interactions with patients, this review reveals that the experimental literature is still quite limited. Moreover, so far, it has yielded conflicting results.

**Figure 3.**
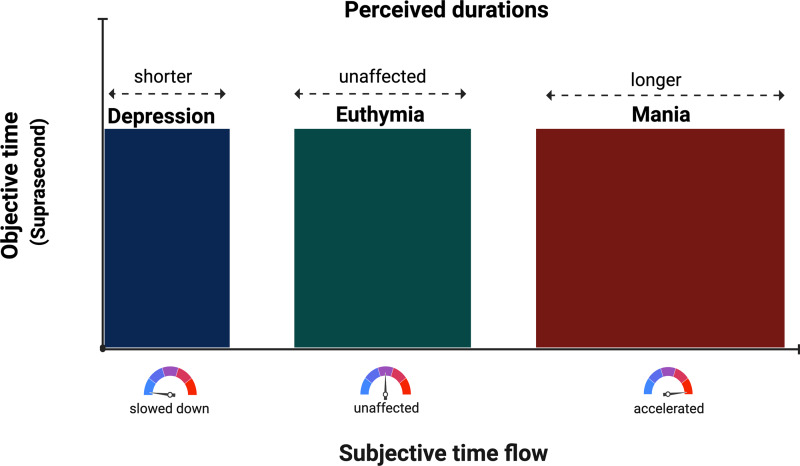
Graphical representation of main results. During depressive states, patients tend to estimate supra-second durations (i.e. objective time) as shorter (i.e. perceived duration) due to slowed-down subjective time flow. During manic states, patients tend to estimate supra-second durations as longer due to accelerated subjective time flow. During euthymic states, perceived durations and subjective time flow of patients tend to be more closely related to objective durations.

Within sub-second temporal ranges, the subjects’ performance in explicit and implicit timing mostly aligns with controls. Notably, these findings appeared coherent. However, they are very few and almost none of them focused on patients with BD during manic states. When considering manic BD patients, their sub-second explicit motor timing is altered (i.e. increased variability and a faster tapping rate in euthymic and manic BD patients) (Bolbecker *et al*., 2011) and so is their implicit perceptual timing (i.e. they have less adaptive eyeblink time-conditioned responses) (Bolbecker *et al*., 2009). As for supra-second temporal spans, the most consistent findings involve BD patients’ tendency towards overestimation of duration during manic states and towards underestimation during depressive states (see Fig. 3). Overall, this tendency characterises both perceptual and motor explicit timing. To this date, supra-second implicit time perception has not been investigated. Moreover, only two studies focused on the minute temporal range but they did not come to unanimous conclusions.

Studies show notable variations in both gender and age distribution of participants. For instance, the percentage of female participants ranges from 43.3% (Liu *et al*., 2022) to as high as 81% (Oyanadel and Buela-Casal, 2014). In terms of age, the sample in Liu et al. ( 2022) includes younger participants with a mean age of around 26 years, while studies such as Mahlberg et al. (2008) and Bschor et al. (2004) focus on older populations, with mean ages exceeding 45 years. These differences in gender and age distributions could potentially affect the generalizability of findings. Indeed, gender may influence clinical presentations, and age may reflect different stages of illness and treatment histories, which may impact the interpretation of perceptual outcomes across studies.

While the characteristics of clinical episodes affect the time perception skills of BD patients overall, only two studies controlled for the presence of psychotic features and found no significant associations (Bolbecker *et al*., 2009; Bolbecker *et al*., 2011; Ryu *et al*., 2015; Arrouet *et al*., 2022; Ciullo *et al*., 2022). Moreover, while patients with schizophrenia were impaired in a temporal order judgement task, this was not the case with BD patients, who performed in the range between controls and schizophrenic patients (Arrouet *et al*., 2022). Similarly, implicit predictive timing was found to be impaired in schizophrenia (Ciullo *et al*., 2018a), but it was not altered in BD patients (Ciullo *et al*., 2022). These results suggest that the alteration of time may be linked with the expression of psychotic symptoms. In line with this hypothesis, a recent meta-analysis of six studies suggested a potential relationship between positive symptoms and altered time perception; this also suggests that psychosis might be associated with the overestimation of timing intervals (Ueda *et al*., 2018). Given these premises, it is possible that time perception varies depending on the severity of bipolar symptoms, with more pronounced alterations potentially being linked to the expression of psychotic features or fluctuations in mood states. However, future research must explore the role of psychotic features in the time perception of BD patients, accounting for cognitive impairments as they play a significant role in both psychosis and timing alterations.

Most studies mentioned in this review did not explore the relationship between timing and cognitive functions. However, the few existing results suggest that time perception alterations may be linked with broader cognitive deficits. This underscores the importance of assessing cognitive functions when conducting studies on time perception in psychiatric disorders. The perception of time is not an isolated function; rather, it is intricately connected with multiple other processes in our brain; for example, supra-second timing is connected with attention and working memory skills (Grondin, 2010). Interestingly, in individuals with schizophrenia, the disruption of time perception occurs independently of broader cognitive impairments (Ciullo *et al*., 2016).

As regards the impact of medications, while the effects of pharmacological agents on time perception are often discussed, no significant results emerged from studies included in the review (Bolbecker *et al*., 2009; Bolbecker *et al*., 2011; Ryu *et al*., 2015; Arrouet *et al*., 2022; Ciullo *et al*., 2022; Liu *et al*., 2022). Several other studies suggest that certain drugs, especially those acting on the dopaminergic system, can influence temporal perception. For instance, dopamine antagonists, such as antipsychotics, have been shown to slow the internal clock (Escelsior *et al*., 2023). Preclinical evidence also points to valproic acid as a cause of deficits in seconds-to-minutes temporal processing, as demonstrated in animal models (Acosta *et al*., 2018). Lithium has been found to have no significant effects on time perception within short intervals (Kolk *et al*., 1993), while other substances like imipramine impair the estimation of longer intervals (Wittenborn *et al*., 1976), and diazepam disrupts the estimation of intervals around 10 s (Unrug-Neervoort *et al*., 1992). Additionally, bromazepam has been shown to increase error in time judgements within the 4–9 s range (Silva *et al*., 2022). Despite these findings, the available literature remains sparse and fragmented, especially when considering shorter temporal intervals.

Several limitations should be acknowledged when interpreting the results of this review. First, given the limited number of studies included, the sample analysed was both small and heterogeneous. This restriction could introduce potential confounds, such as small effect sizes in results and a lack of significant differences due to merging for clinical phases or types of BD (type I/type II). Studies exploring shorter temporal spans have suggested no differences between BD patients and controls; however, these studies did not differentiate patients based on their clinical states. Another significant limitation identified in the reviewed studies is the lack of robust longitudinal research employing experimental methodologies to assess the temporality shifts in BD patients based on specific individual clinical phases. Indeed, we found that only three studies (Mezey and Knight, 1965; Elsass *et al*., 1979; Tysk, 1985) conducted longitudinal assessments, but none of these met the criteria for this systematic review (see the Supplementary Materials). However, these studies are either anecdotal, characterised by a very small sample size, or have unspecified follow-up periods. Collectively, they yield inconclusive results, not allowing to formulate a hypothesis supported by experimental evidence regarding the state or trait nature of potential alterations in temporal perception. At the same time, the main significant shortcoming is that the available results do not delve into the neurobiological correlates of timing performances and, to our knowledge, there are no available articles that directly investigate the neurobiological mechanisms associated with time perception. In their study on implicit timing, Bolbecker et al. (2009) posited a link between impaired sub-second time processing and alterations in cerebellar circuitry in BD patients. Future research should focus on the multimodal neuroimaging of the cerebellum to explore its association with timing alterations and its potential therapeutic target, as has been suggested for schizophrenia (Escelsior and Belvederi Murri, 2019; Escelsior *et al*., 2019). The study of the neurobiological correlates of temporality in BD patients must also consider the investigation of the neurotransmitter systems involved in time perception. Indeed, several neurotransmitter systems contribute to this function, most notably the dopaminergic (DA) system (Escelsior *et al*., 2023), and it is known that BD is widely characterised by abnormalities in DA neurotransmission (Grande *et al*., 2016).

## Conclusions

The existing literature suggests that BD patients are mostly similar to controls in time perception within sub-second temporal intervals. In turn, manic BD patients tend to overestimate time and depressed BD patients tend to underestimate it when dealing with longer temporal spans. However, due to the multifaceted nature of time perception, not enough studies on the topic exist to draw specific conclusions. Moreover, the underlying neurobiological mechanisms of time perception in BD patients remain obscure. Based on this literature review, future studies on the topic should carefully consider the clinical episodes of patients with BD along with their cognitive skills. Given the current lack of reliable biomarkers in psychiatry that bridge neurobiological mechanisms and clinical symptoms, time perception should be further investigated as it could serve as a valuable intermediate phenotype. This has the potential to enhance research, diagnosis, and therapeutic interventions in BD, ultimately contributing to more effective public health strategies.

## Supporting information

Escelsior et al. supplementary material Escelsior et al. supplementary material

## Supplementary material

The supplementary material for this article can be found at https://doi.org/10.1017/neu.2024.57.
