# Supplementary material for: Time perception in bipolar disorder: a systematic review
Source: Acta Neuropsychiatr. 2025 Jan 23;37:e5. doi: 10.1017/neu.2024.57 (PMC13130252; doi:10.1017/neu.2024.57)
Supplement: Escelsior et al. supplementary material [file S0924270824000577sup001.docx]

**Supplementary materials**

**Temporal perception in bipolar disorder: a systematic review**

Andrea Escelsior^1,2†^*, Maria Bianca Amadeo^3†^, Alberto Inuggi^2^, Yara Masshala^2^, Margherita Guzzetti^2^, Beatriz Pereira da Silva^2^, Monica Gori^3^, Mario Amore^2^, Gianluca Serafini^1,2^

^1^IRCCS Ospedale Policlinico San Martino, Genoa, Italy

^2^Department of Neuroscience, Rehabilitation, Ophthalmology, Genetics, Maternal and Child Health (DINOGMI), Section of Psychiatry, University of Genoa

^3^U-VIP Unit for Visually Impaired People, Fondazione Istituto Italiano di Tecnologia, Genoa, Italy.

^†^ These authors contributed equally

*Table S1. Results of relevant literature.*

| **Study** | **Language** | **Control group** | **Diagnostic criteria specified** | **Experimental setting** | **Relevant timing assessment** | **Adequate statistical information** | **Inclusion** | **Description** |
| --- | --- | --- | --- | --- | --- | --- | --- | --- |
| (Fouks et al., 1961) | Fre | n.i. | n.i. | n.i. | n.i. | n.i. | No |  |
| (Hishiyama and Fukasawa, 1964) | Jap | n.i. | n.i. | n.i. | n.i. | n.i. | No |  |
| (Mezey and Knight, 1965) | Eng | Yes | No | Yes | No | Yes | No | The study explores time judgment and reproduction in hypomanic patients, comparing their assessments during hypomania and after recovery, and depressed patients. |
| (Stein, 1977) | Eng | No | No | Yes | No | No | No | The study explores the temporal errors during music perception in mania. |
| (Elsass et al., 1979) | Eng | Yes | No | Yes | No | Yes | No | The study evaluates time estimation and reproduction in bipolar disorder, with findings indicating a subjective acceleration of time. |
| (Nielzen and Cesarec, 1982) | Eng | Yes | No | No | No | Yes | No | The study investigated the emotional experience associated with music in mania. |
| (Tysk, 1984) | Eng | Yes | Yes  (DSM-III) | Yes | Yes  (TET, TPT, MA) | Yes | Yes | Described in the review. |
| (Kut'ko et al., 1985) | Rus | n.i. | n.i. | n.i. | n.i. | n.i. | No |  |
| (Tysk, 1985) | Eng | No | Yes  (DSM-III) | Yes | No | Yes | No | The study examines longitudinal time estimation changes in a diverse affective disorders’ cohort. |
| (Stein, 1988) | Eng | No | No | No | No | No | No | In a Letter to the Editor, the authors examine musical tempo misperceptions in manic subjects. |
| (Iarovitskiĭ and Baturin, 1991) | Rus | n.i. | n.i. | n.i. | n.i. | n.i. | No |  |
| (Nosachev, 1991b) | Rus | n.i. | n.i. | n.i. | n.i. | n.i. | No |  |
| (Nosachev, 1991a) | Eng | Yes | No | Yes | No | No | No | The study explores time perception in subjects with manic-depressive and schizophrenic illnesses. |
| (Bschor et al., 2004) | Eng | Yes | Yes  (DSM-IV) | Yes | Yes  (TET, TPT) | Yes | Yes | Described in the review. |
| (Penney et al., 2005) | Eng | No | N/A | Yes | Yes  (TBT) | Yes | No | The study investigates time estimation differences among participants at high genetic risk for schizophrenia, major affective disorders, and healthy controls. |
| (Mahlberg et al., 2008) | Eng | Yes | Yes  (DSM-IV) | Yes | Yes  (CT) | Yes | Yes | Described in the review. |
| (Bolbecker et al., 2009) | Eng | Yes | Yes  (DSM-IV) | Yes | Yes  (EBR) | Yes | Yes | Described in the review. |
| (Zhao et al., 2010) | Chi | n.i. | n.i. | n.i. | n.i. | n.i. | No |  |
| (Bolbecker et al., 2011) | Eng | Yes | Yes  (DSM-IV) | Yes | Yes  (PFTT) | Yes | Yes | Described in the review. |
| (Gruber et al., 2012) | Eng | Yes | Yes  (DSM-IV-TR) | No | No | Yes | No | Two studies on emotional time perspective and (1) correlations found between hypomanic traits in healthy subjects (2) differences observed between bipolar disorder patients and healthy controls. |
| (Bolbecker et al., 2014) | Eng | Yes | Yes  (DSM-IV) | Yes | Yes  (TBT) | Yes | Yes | Described in the review. |
| (Oyanadel and Buela-Casal, 2014) | Eng | Yes | Yes  (DSM-IV-TR) | Quasi-experimental | Yes  (TET, TPT) | Yes | Yes | Described in the review. |
| (Ryu et al., 2015) | Eng | Yes | Yes  (DSM-IV) | Yes | Yes  (TET-I, TPT-I) | Yes | Yes | Described in the review. |
| (Glazer et al., 2019) | Eng | No | N/A | Yes | Yes | Yes | No | The study investigates timing and ERP correlates of hypomanic personality traits and unipolar depressive symptom proneness in healthy subjects. |
| (Ciullo et al., 2022) | Eng | Yes | Yes  (DSM-5) | Yes | Yes  (TBT, TDT, PT, TPT) | Yes | Yes | Described in the review. |
| (Karaytug et al., 2022) | Eng | Yes | Yes  (DSM-V) | No | No | Yes | No | The study assesses the relationship between chronotype and suicide in bipolar disorder through time perspective. |
| (Liu et al., 2022) | Eng | Yes | Yes  (DSM-V) | Yes | Yes  (TBT) | Yes | Yes | Described in the review. |
| (Arrouet et al., 2022) | Eng | Yes | Yes (DSM-V) | Yes | Yes  (TOJ) | Yes | Yes | Described in the review. |
| (Weintraub et al., 2023) | Eng | No | Yes  (DSM-V) | Yes | No | Yes | No | The study analyzes digital phenotyping of spontaneous speech and its link with depressive symptoms in at-risk youth, finding a correlation with time orientation words. |

**Legend**: **CT,** Chronotest; **N/A**, Not Applicable; **n.i.** Not investigable; **EBR** Eye blink reflex; **MA**, Metronome adjustment; **PT**, Predictive timing; **TBT¸** Temporal bisection task; **TDT,** temporal discrimination task; **TET-I**, Time estimation and reproduction tasks using the IAPS figures; **TET**, Temporal estimation task; **PFTT**, Paced finger-tapping tasks; **TOJ**, Temporal order judgment task; **TPT**, Temporal production /reproduction task; **TPT-I**, Time production/reproduction tasks using the IAPS figures.

*Table S2. Summary of Results from the Methodological Quality Assessment of Cross-Sectional Studies with the Appraisal Tool for Cross-Sectional Studies Scale.*

| Study ID | | Clear Aims and Objectives | Study Design Appropriate? | Sample Size Justified? | Target Population Clearly Defined? | Appropriate population? | Selection Process | Appropriate Risk Factors and Outcomes? | Measurement of Outcomes | Statistical Analysis | Basic Data Adequately Described? | Methods Described in Details? | Non-response Bias? | Internally Consistent Results? | Results of Analyses Presented? | Conclusions Justified by Results? | Limitations Discussed? | Conflicts of Interest? | Ethical Approval and Consent? | **Overall Quality** |
| --- | --- | --- | --- | --- | --- | --- | --- | --- | --- | --- | --- | --- | --- | --- | --- | --- | --- | --- | --- | --- |
| Arrouet et al., 2022 | |  |  |  |  |  |  |  |  |  |  |  | N/A |  |  |  |  |  |  |  |
| Ciullo et al., 2022 | |  |  |  |  |  |  |  |  |  |  |  | N/A |  |  |  |  |  |  |  |
| Liu et al., 2021 | |  |  |  |  |  |  |  |  |  |  |  | N/A |  |  |  |  |  |  |  |
| Ryu et al., 2015 | |  |  |  |  |  |  |  |  |  |  |  | N/A |  |  |  |  |  |  |  |
| Bolbecker et al., 2014 | |  |  |  |  |  |  |  |  |  |  |  | N/A |  |  |  |  |  |  |  |
| Oyanadel et al., 2014 | |  |  |  |  |  |  |  |  |  |  |  | N/A |  |  |  |  |  |  |  |
| Bolbecker et al., 2011 | |  |  |  |  |  |  |  |  |  |  |  | N/A |  |  |  |  |  |  |  |
| Bolbecker et al., 2009 | |  |  |  |  |  |  |  |  |  |  |  | N/A |  |  |  |  |  |  |  |
| Mahlberg et al., 2008 | |  |  |  |  |  |  |  |  |  |  |  | N/A |  |  |  |  |  |  |  |
| Bschor et al., 2004 | |  |  |  |  |  |  |  |  |  |  |  | N/A |  |  |  |  |  |  |  |
| Tysk, 1984 | |  |  |  |  |  |  |  |  |  |  |  | N/A |  |  |  |  |  |  |  |
|  | High quality/Low risk of bias | | |  |  |  |  |  |  |  |  |  |  |  |  |  |  |  |  |  |
|  | Moderate quality/ Risk of bias | | |  |  |  |  |  |  |  |  |  |  |  |  |  |  |  |  |  |
|  | Low quality/ High risk of bias | | |  |  |  |  |  |  |  |  |  |  |  |  |  |  |  |  |  |

**Details about psychopathological correlates of temporal perception**

When evaluating explicit perception of time, Ciullo et al. (Ciullo et al., 2022) considered different psychopathological indices and added them in a forward stepwise linear regression analysis to identify possible predictors of the timing performance. Specifically, they evaluated the occurrence of depressive, manic, and hypomanic episodes, the total score on the Young Mania Rating Scale (YMRS) (Young et al., 1978), the total score on the Hamilton Depression Rating Scale (HAM-D) (Hamilton, 1960), and sub-scores from the Positive and Negative Syndrome Scale (PANSS) (Kay et al., 1987) which includes Positive, Negative, Cognitive, Excitement/Hostility, and Emotional Discomfort dimensions. Additionally, they considered whether patients were on antipsychotic medications, factoring in the chlorpromazine equivalents (CPZE, *n =* 20). Of these psychopathological variables, the number of previous depressive episodes was the sole predictor of BD patients’ precision in the temporal reproduction task – the only task where patients were observed to differ from the control group.

Liu et al. (Liu et al., 2022) examined the correlation between accuracy and precision in the temporal bisection task (encompassing both sub-second and supra-second explicit perception of time) and the HAM-D score. They found no significant relationships.

No significant results were also obtained by Arrouet et al. (Arrouet et al., 2022) when investigating the correlation between participants’ CPZE medication and the explicit performance at the temporal order judgment task.

Ryu et al. (Ryu et al., 2015) segmented their sample based on the manic and euthymic states to investigate potential psychopathological correlates. They assessed performance in both supra-second estimation and reproduction tasks, which are explicit measures of time perception. In their analysis, they considered scores from the Brief Psychiatric Rating Scale (BPRS) (Pull and Overall, 1977), the YMRS, the Montgomery–Åsberg Depression Scale (MADRS) (Montgomery and Åsberg, 1979), the Clinical Global Impression (CGI), and the level of antipsychotic medications determined by CPZE. Interestingly, euthymic patients were similar to control individuals, while temporal judgments of emotional pictures showed a different pattern for manic patients. As for the clinical variables, significant correlations emerged only for the manic group between performance at estimation task and the manic and illness symptom severity, as measured by the YMRS and the CGI.

Bolbecker et al. (Bolbecker et al., 2014) investigated the potential correlation between accuracy and precision in an explicit sub-second temporal bisection task and the scores on the YMRS and MADRS for BD groups, both with and without psychotic features. Their findings did not yield any significant results.

Bolbecker et al. (2011) (Bolbecker et al., 2011) failed to find significant differences between manic (*n =* 17) and euthymic (*n =* 25) subjects on a finger-tapping performance (explicit supra-second task of perception of time). Moreover, their behavioral data did not correlate with YMRS or MADRS scores. They also evaluated possible pharmacological effects by classify BD patients differently based on the medication. First, they considered separately individuals assuming typical or atypical antipsychotics (n =25), other psychotropic drugs (*n =* 9), and unmedicated individuals (*n =* 8). Subsequently, they categorized patients based on use of atypical antipsychotic drug use (*n =* 22), lithium use (*n =* 8), and any psychotropic medication including antipsychotics versus unmedicated participants (*n =* 34). None of these approaches led to significant results.

Bolbecker et al. (Bolbecker et al., 2009) investigated the presence and temporal latency of conditioning responses to auditory stimuli, which is an implicit sub-second timing task. They considered the mood state (i.e., manic, euthymic, and mixed) and pharmacological data of participants. Their findings indicated that the timing deficit was predominantly observed in patients with a mixed episode. However, there was no correlation between conditioning results and either YMRS or MADRS scores. When comparing unmedicated patients (*n =* 7) with age-matched controls, they found similar results as with the entire BD group. Moreover, CPZE (*n =* 10) and valproate (*n =* 7) dosages were not significantly correlated with percent or peak latency of the conditioned responses.

Mahlberg et al. (Mahlberg et al., 2008) separated the BD sample in patients in either manic or depressive state, and reported differences between the two phases of the illness in a temporal reproduction task (explicit supra-second task). However, when they explored the influence of illness severity by correlating performance with scores from either the HAM-D or the Bech–Rafaelsen Mania Scale (BRMS) (Bech et al., 1978), no significant results emerged.

Bschor et al. (Bschor et al., 2004) investigated the effects of the clinical and psychopathological characteristics by conducting analyses on the duration of disorder, number of prior episodes, duration of the current episode, and total score at either the HAM-D or the BRMS. In their association analysis, they found that poorer performance was linked with the number of previous episodes, but this relationship was exclusive to the depressed participants. Notably, results from both supra-second explicit temporal estimation and production tasks did not correlate with any clinical or psychopathological variables.

Tysk (Tysk, 1984) reported significant differences between manic (*n* =11), depressed (*n* = 8) and in remission (*n* = 9) patients in various tasks of explicit temporal perception. Specifically, in both perceptual (i.e., metronome adjustment and verbal estimation) and motor (i.e., production) timing, manic patients tended to overestimate time, depressed patients to underestimate it, and patients in remission behaved similar to controls. Nonetheless, it is important to note that due to small sample size BD patients with a depressive episode were analyzed together with patients with major depression.

**Details about cognitive correlates of temporal perception**

Ciullo et al. (Ciullo et al., 2022) included in the analyses two neuropsychological measures. They administered the Delayed Item Recognition task (DIR) (Ciullo et al., 2018, Ciullo et al., 2022) to examine non-verbal and non-visuo-spatial working memory (WM). Additionally, they employed the Trail Making Test part-A (TMT-A) (Reitan, 1992) to assess processing speed, WM, and set-shifting performances. In a forward stepwise linear regression analysis assessing potential predictors of precision in the temporal reproduction task, the accuracy of the Delayed Item Recognition task explained, together with the number of previous major depressive episode, the 20% of variance. Notably, performance on the Delayed Item Recognition task, which evaluates WM, showed no difference between the two groups.

Bolbecker et al. (Bolbecker et al., 2014) correlated the results at the temporal bisection task (explicit sub-second task time perception) with the QI information derived from the Wechsler Abbreviated Scale of Intelligence (WASI) (Wechsler, 1999) and found no relationship between QI and either temporal precision or accuracy.

While investigating supra-second temporal estimation and production, Bschor et al. (2004) (Bschor et al., 2004) assessed perceptual, executive, and motor speed with the TMT part-A and showed that a retardation was associated with an overestimation of time only in BD individuals with a manic episode.

**Neurobiological assumptions underlying temporal perception**

No studies involving BD patients investigated the neurobiological correlates of temporal perception. Based on the existing literature implicating a role of the cerebellum in eyeblink conditioning responses, Bolbecker et al. (Bolbecker et al., 2009) suggested that the inability of the BD group to adaptively delay their conditioned blink responses was consistent with cerebellar functional abnormalities. Thus, they hypothesized that cerebellar dysfunction may contribute to poor temporal coordination as revealed by the eyeblink conditioning task.

Bolbecker et al. (Bolbecker et al., 2011) applied to the results of a finger-tapping task a mathematical model, namely the Wing–Kristofferson model (Wing and Kristofferson, 1973a, Wing and Kristofferson, 1973b), to indirectly disentangle whether the source of timing variability was due to the central timekeeper or to a delay between neural command and the execution of the movement. By decomposing the timing variance during the continuation phase (i.e., continuing tapping at the same rhythm without the stimulus), it emerged that the BD group had higher variability in the internal timekeeping system but similar motor implementation variability compared to controls. Within the BD group, differences between manic and euthymic patients were not significant.

**References**

**Arrouet A, Polgári P, Giersch A and Joos E** (2022) Temporal Order Judgments in Schizophrenia and Bipolar Disorders–Explicit and Implicit Measures. *Timing & Time Perception,* **11,** 362-385.

**Bech P, Rafaelsen O, Kramp P and Bolwig T** (1978) The mania rating scale: scale construction and inter-observer agreement. *Neuropharmacology*.

**Bolbecker AR, Hong SL, Kent JS, Forsyth JK, Klaunig MJ, Lazar EK, O'donnell BF and Hetrick WP** (2011) Paced finger-tapping abnormalities in bipolar disorder indicate timing dysfunction. *Bipolar Disord,* **13,** 99-110.

**Bolbecker AR, Mehta CS, Edwards CR, Steinmetz JE, O'donnell BF and Hetrick WP** (2009) Eye-blink conditioning deficits indicate temporal processing abnormalities in schizophrenia. *Schizophr Res,* **111,** 182-91.

**Bolbecker AR, Westfall DR, Howell JM, Lackner RJ, Carroll CA, O'donnell BF and Hetrick WP** (2014) Increased timing variability in schizophrenia and bipolar disorder. *PLoS One,* **9,** e97964.

**Bschor T, Ising M, Bauer M, Lewitzka U, Skerstupeit M, Muller-Oerlinghausen B and Baethge C** (2004) Time experience and time judgment in major depression, mania and healthy subjects. A controlled study of 93 subjects. *Acta Psychiatr Scand,* **109,** 222-9.

**Ciullo V, Piras F, Banaj N, Vecchio D, Piras F, Sani G, Ducci G and Spalletta G** (2022) Internal clock variability, mood swings and working memory in bipolar disorder. *J Affect Disord,* **315,** 48-56.

**Ciullo V, Vecchio D, Gili T, Spalletta G and Piras F** (2018) Segregation of Brain Structural Networks Supports Spatio-Temporal Predictive Processing. *Front Hum Neurosci,* **12,** 212.

**Elsass P, Mellerup ET, Rafaelsen OJ and Theilgaard A** (1979) Lithium effects on time estimation and mood in manic-melancholic patients. A study of diurnal variations. *Acta Psychiatr Scand,* **60,** 263-71.

**Fouks M, Laine, Perivier, Mathis, Bouchey and Riout** (1961) *Psychopathologie de la temporalite et de la festivite maniaque,* Paris, France, Masson Editeur.

**Glazer JE, Kelley NJ, Pornpattananangkul N and Nusslock R** (2019) Hypomania and depression associated with distinct neural activity for immediate and future rewards. *Psychophysiology,* **56,** e13301.

**Gruber J, Cunningham WA, Kirkland T and Hay AC** (2012) Feeling stuck in the present? Mania proneness and history associated with present-oriented time perspective. *Emotion,* **12,** 13-7.

**Hamilton M** (1960) A rating scale for depression. *J Neurol Neurosurg Psychiatry,* **23,** 56-62.

**Hishiyama T and Fukasawa F** (1964) An analysis of mental states in the experimental conditions of an estimation of lapse of time. Functional analysis of mental disorders by response performance. *Seishin Shinkeigaku Zasshi= Psychiatria et Neurologia Japonica,* **66,** 468-477.

**Iarovitskiĭ V and Baturin V** (1991) Reproduction of the minute time interval in depression in patients with schizophrenia and manic-depressive psychosis. *Zhurnal Nevropatologii i Psikhiatrii Imeni SS Korsakova (Moscow, Russia: 1952),* **91,** 112-114.

**Karaytug MO, Tamam L, Demirkol ME, Namli Z, Gurbuz M, Yesiloglu C and Eris Davut O** (2022) The Mediating Role of Time Perspective in the Relationship between Chronotype and Suicide in Bipolar Disorder. *Behav Sci (Basel),* **12**.

**Kay SR, Fiszbein A and Opler LA** (1987) The positive and negative syndrome scale (PANSS) for schizophrenia. *Schizophr Bull,* **13,** 261-76.

**Kut'ko I, Stefanovskiĭ V, Bukreev V and Shestopalova L** (1985) Time perception in depressive states. *Vrachebnoe Delo***,** 99-101.

**Liu P, Guo H, Ma R, Liu S, Wang X, Zhao K, Tan Y, Tan S, Yang F and Wang Z** (2022) Identifying the difference in time perception between major depressive disorder and bipolar depression through a temporal bisection task. *PLoS One,* **17,** e0277076.

**Mahlberg R, Kienast T, Bschor T and Adli M** (2008) Evaluation of time memory in acutely depressed patients, manic patients, and healthy controls using a time reproduction task. *Eur Psychiatry,* **23,** 430-3.

**Mezey AG and Knight EJ** (1965) Time Sense in Hypomanic Illness. *Arch Gen Psychiatry,* **12,** 184-6.

**Montgomery SA and Åsberg M** (1979) A new depression scale designed to be sensitive to change. *The British journal of psychiatry,* **134,** 382-389.

**Nielzen S and Cesarec Z** (1982) Aspects of tempo and perception of music in mania. *Acta Psychiatr Scand,* **65,** 81-5.

**Nosachev G** (1991a) Time perception and surviving by depressive patients with manic-depressive psychosis and attack-like schizophrenia. *Žurnal nevropatologii i psihiatrii im. SS Korsakova,* **91,** 114-117.

**Nosachev G** (1991b) Time perception by patients with depression in manic-depressive psychosis and recurrent schizophrenia. *Zhurnal Nevropatologii i Psikhiatrii Imeni SS Korsakova (Moscow, Russia: 1952),* **91,** 114-117.

**Oyanadel C and Buela-Casal G** (2014) Time perception and psychopathology: Influence of time perspective on quality of life of severe mental illness. *Actas Esp Psiquiatr,* **42,** 99-107.

**Penney TB, Meck WH, Roberts SA, Gibbon J and Erlenmeyer-Kimling L** (2005) Interval-timing deficits in individuals at high risk for schizophrenia. *Brain Cogn,* **58,** 109-18.

**Pull CB and Overall JE** (1977) Adequacy of the Brief Psychiatric Rating Scale for distinguishing lesser forms of psychopathology. *Psychol Rep,* **40,** 167-73.

**Reitan R** (1992) Trail making test: Manual for administration and scoring: Reitan Neuropsychology Laboratory. *Back to cited text*.

**Ryu V, Kook S, Lee SJ, Ha K and Cho HS** (2015) Effects of emotional stimuli on time perception in manic and euthymic patients with bipolar disorder. *Prog Neuropsychopharmacol Biol Psychiatry,* **56,** 39-45.

**Stein J** (1977) Tempo errors and mania. *Am J Psychiatry,* **134,** 454-6.

**Stein J** (1988) Manic tempo misperceptions. *Biol Psychiatry,* **24,** 366-8.

**Tysk L** (1984) Time perception and affective disorders. *Percept Mot Skills,* **58,** 455-64.

**Tysk L** (1985) Longitudinal changes in time estimation in affective disorders: a preliminary study. *Percept Mot Skills,* **60,** 179-88.

**Wechsler D** (1999) Wechsler abbreviated scale of intelligence.

**Weintraub MJ, Posta F, Ichinose MC, Arevian AC and Miklowitz DJ** (2023) Word usage in spontaneous speech as a predictor of depressive symptoms among youth at high risk for mood disorders. *J Affect Disord,* **323,** 675-678.

**Wing AM and Kristofferson A** (1973a) The timing of interresponse intervals. *Perception & Psychophysics,* **13,** 455-460.

**Wing AM and Kristofferson AB** (1973b) Response delays and the timing of discrete motor responses. *Perception & Psychophysics,* **14,** 5-12.

**Young RC, Biggs JT, Ziegler VE and Meyer DA** (1978) A rating scale for mania: reliability, validity and sensitivity. *Br J Psychiatry,* **133,** 429-35.

**Zhao Q, Ji Y, Wang K, Zhang L, Liu P and Jiang Y** (2010) Time perception in depressed and manic patients. *Zhonghua yi xue za zhi,* **90,** 332-336.
